# Our Journal

**Published:** 1871-01

**Authors:** 


### PART III.

### Editorials, Reviews, Items and News.

### OUR JOURNAL.

In making our *debut* before the readers of the Companion, we greet them with a cordial, warm-hearted Salutatory. Having thus tendered them that editorial courtesy, which, we trust, is inspired by the spirit of good will we bear them and the noble calling they have espoused, it is still our duty, as well as pleasure, to bring before them the object we have in view in the publication of the Companion—the first number of which we now have the gratification of presenting for their inspection.

What, then, is the true mission of the Companion? Shall it be for good, or shall it be the harbinger of evil to the profession? The aims and objects of a Medical Journal cannot be fully elucidated in one editorial—particularly when questions unknown to us in the future, must arise to unfold new ideas and shape our action. This much, however, we promise ourselves and readers, that we shall earnestly do our best, and in doing this shall be guided by one brief rule—"Do Right."

We shall not hesitate to frankly give our opinions upon all topics coming legitimately within our sphere as medical journalists. If thus speaking, we shall meet the approval of friends, we shall be pleased aud gratified; if, on the other hand, we should arouse enmity by adherence to principle and truth, we shall deplore, but not deprecate the fact. Enemies, when made so by honest action; when merely the out-cropping of bad motives, in opposition to truth and right—are not a misfortune. They show the pathway of duty the plainer. For these all good men have a use, as incentives to higher endeavors—a stimulus to a nobler life.

Surely no medical gentleman of the State, who has the interest of his profession, or the welfare of humanity, at heart, can lament the appearance of a Medical Journal in his midst. Should he do so, the public ought to suspect his inability to keep step to the march of progressive science, when that science, with its untold treasures of hidden gems, lie unexhumed; when day by day, new discoveries spring, Minerva-like, from the brains of its toiling votaries, to fit them for the combat against that ruthless destroyer—disease.

The true Physician must ever be an earnest student; and the best friend he can secure is a Medical Journal, which, from time to time, amid the difficulties and perplexities of professional duties, shall present modes of practice and tried remedies, of practical value, as aids to professional efficiency. He who does not feel the weight of responsibility entrusted to his keeping, when the precious lives of kindred and friends hang suspended upon his efforts, is unworthy the name of physician; and the public, often uninformed and unsuspecting, as to the science of medicine, and the true qualifications of the physician, have a right to all the benefits true science can bestow.

Our object, then, will be to place monthly before our readers, short, pointed, practical selections from both our home and foreign medical literature. Theories—while serving a good purpose, in many instances, and imposing and useless in others—shall give place to the wants of the busy practitioner, who demands the known rather than the purely speculative. We shall strive to gather from all sources, whatever we may deem strictly valuable to the active physician, in the various departments of medical science, as developed in home and foreign periodicals, standard works, and direct original matter from physicians.

Our field of labor being almost wholly unoccupied—there being no Medical Journal in Georgia, South Carolina, Alabama or Mississippi—we can jostle the interest of no one, while the plain, straight-forward path which we shall persistently pursue, will bring legitimate results which cannot but prove beneficial to all.

To enlist the active co-operation of the good and true of our professional brethren, we shall endeavor to make it the exponent of the truths of our science that have been discovered in the past, or shall be developed in the future. There shall be no winking at the sacrilegious acts of innovators and imposters, who, under the plea of necessity, and the credulity of the masses, proclaim themselves in the public press, as the only skilled proficients of our art—as specialists. And regarding the ethics as the embodiment of wisdom, founded upon a true morality—the only basis of honor and religion—which bids men, in every sphere of life, "to do to others as you would that they should do to you," we shall, to the best of our ability, maintain and defend them. A faithful adherence to these ethical principles can alone advance the cause of medical science, the well-being of its members, and afford protection to the public against the wiles of the charlatan and the schemes of the negative medical demagogue—whose actions are so often the hypocritical expressions of refinement, religion and cultivation, for selfish purposes and local advantages.

As almost every physician can from his own experience, impart some valuable information for the general good, we most earnestly solicit all to send us for publication any matter of practical importance, written as concisely as possible.

To our exchanges we will say, that from the foregoing plan it will be seen that it is our design, as far as possible, to gather, as it were, the cream of each periodical coming to our hands, and lay it before our readers. In doing this, each selection shall be accompanied by the name of the author, and that of the journal from which it shall be taken—thus making our exchanges familiar to the profession of our section.

Having thus stated the plan and objects of our journal, we ask our friends everywhere to extend to it and us their active, zealous co-operation, in order to secure for it a circulation commensurate with the importance demanded by a science so progressive and humane, as Medicine. Let our Journal, by your aid, be the instrument of imparting medical truths to every physician of our section, that from this knowledge, gathered from a thousand pens, they may confer happiness, with health, upon those for whose welfare they labor.

The typographical execution of the Companion speaks for itself. It could not but be of the highest order of that art, under the supervision of the enterprising proprietor of the "Franklin Steam Printing and Publishing House," of this city. We can confidently assure our readers there will be no "falling away," in any respect, in the hands of Mr. J. J. Toon, whose name is ample guaranty of the success and permanency of the Georgia Medical Companion.

